# Changing Body Representation Through Full Body Ownership Illusions Might Foster Motor Rehabilitation Outcome in Patients With Stroke

**DOI:** 10.3389/fpsyg.2020.01962

**Published:** 2020-08-21

**Authors:** Marta Matamala-Gomez, Clelia Malighetti, Pietro Cipresso, Elisa Pedroli, Olivia Realdon, Fabrizia Mantovani, Giuseppe Riva

**Affiliations:** ^1^“Riccardo Massa” Department of Human Sciences for Education, University of Milano-Bicocca, Milan, Italy; ^2^Department of Psychology, Catholic University of Milan, Milan, Italy; ^3^Applied Technology for Neuro-Psychology Laboratory, Istituto Auxologico Italiano, IRCCS, Milan, Italy; ^4^Faculty of Psychology, eCampus University, Novedrate, Italy

**Keywords:** body representation, body ownership illusions, 360° videos, virtual reality, body schema, neurorehabilitation

## Abstract

How our brain represents our body through the integration of internal and external sensory information so that we can interact with our surrounding environment has become a matter of interest especially in the field of neurorehabilitation. In this regard, there is an increasing interest in the use of multisensory integration techniques—such as the use of body ownership illusions—to modulate distorted body representations after brain damage. In particular, cross-modal illusions such as mirror visual feedback therapy (MVFT) have been widely used for motor rehabilitation. Despite the effectiveness of the MVFT for motor rehabilitation, there are some limitations to fully modify the distorted internal representation of the paretic limb in patients with stroke. A possible explanation for this relies on the physical limitations of the mirror in reproducing upper-limb distortions, which can result in a reduced sense of ownership of the mirrored limb. New digital technologies such as virtual reality (VR) and 360° videos allow researchers to create body ownership illusions by adapting virtual bodies so that they represent specific morphological characteristics including upper-limb distortions. In this manuscript, we present a new rehabilitation approach that employs full virtual body ownership illusions, using a 360° video system, for the assessment and modulation of the internal representation of the affected upper limb in stroke patients. We suggest modifying the internal representation of the upper limb to a normal position before starting motor rehabilitation training.

## Introduction

How our brain represents our body has been a matter of interest in the field of neuropsychology and neuroscience for many years (Lhermitte, 1942). In this regard, the sense of embodiment (or bodily self), that is, the sense of having a body (Gallagher, 2000), emerges from a complex interaction between bottom-up sensory signals and top-down cognitive processes occurring within a body frame (Longo et al., 2008; Tsakiris, 2017). More specifically, the sense of embodiment has been described as composed of several different structurally organized subjective components: (1) ownership, (2) agency, and (3) self-location (Longo et al., 2008; Kilteni et al., 2012a). In fact, one fundamental component of embodiment is the sense of body ownership (Tsakiris, 2017). The sense of body ownership is described as the percept of a body part or entire body belonging to oneself (Ehrsson, 2020). The sense of agency, meaning the sense of being the initiator or the source of the body’s actions, is another fundamental component of embodiment (Gallagher, 2000). In addition, the sense of embodiment is constructed around the first-person pronoun from a conceptual point of view (Gallagher, 2012), where the subject feels self-located inside a physical body (Lenggenhager et al., 2006). The perceptual distinction between what is part of one’s body and what is not is a crucial factor for human perception, action, and cognition (Ehrsson, 2020). Then, the sense of embodiment is multisensory in nature and relies on how all different sensory modalities come together into a coherent percept of an owned body or body part (Ehrsson, 2020).

The representation of embodiment (or bodily self) is typically explained according to two distinct concepts: body image and body schema (Gallagher, 1986). The conscious body image, as Gallagher described it, consists of three different components: first, the perception of the body in the immediate consciousness; second, the cognitive conceptualization of the body that is influenced by the immediate consciousness and the knowledge about the body; and third, the emotions and feelings toward the body that may be generated by conscious or unconscious experiences. Therefore, body image is constructed based on perceptual, cognitive, and emotional components. Body schema, on the other hand, was described by Gallagher as a more organized representation of the body in relation to the environment. More specifically, Gallagher refers to body schema as an active component of body representation, which integrates different body positions and movements in relation to the environment (Gallagher, 1986). Body schema is additionally defined by different authors as a dynamic sensorimotor representation of the body that leads to body perception and body action (Dohle et al., 2004; Haggard and Wolpert, 2005; de Vignemont, 2010; Longo et al., 2010).

Body representation can change due to an injury to the nervous system, resulting in the modification of the body’s internal multisensory interactions (Berlucchi and Aglioti, 2010). It is known that nervous system injuries, as well as mental illnesses, can change the internal representation of the body (Berlucchi and Aglioti, 2010; Leemhuis et al., 2019). Internal representations of the body refer to relatively stable representations of the body and define the body as it usually is (Berlucchi and Aglioti, 2010). Damage in the right hemisphere of the brain, specifically in the temporoparietal and insular areas, can disrupt spatial and body representations, as in spatial neglect (Heilman et al., 2000). Moreover, in amputee or hemiparetic patients, a sensorimotor interruption after the injury, arm amputation, or paresis of a body part can distort the internal body representation, leading to consequences such as phantom limb phenomena or motor anosognosia (denial of the motor deficits commonly observed in patients with right-hemisphere brain damage; Berlucchi and Aglioti, 2010). Brain damage can also lead to distorted body representations that bring about alterations in proprioceptive and kinesthetic signals and in the perception of the peripersonal space (the space around the body; Wallwork et al., 2016). Such sensory alterations after brain damage affect movement planning, preparation, and execution, since motor performance is continuously fostered by sensorimotor loops that constantly update internal predictions about the outcome of a motor command (Wolpert and Ghahramani, 2000). In addition, it has been argued that all these neural interactions occur within a cortical body matrix frame, which can be thought of as a neural network that is implicated in the regulation, control, and protection of the body and the surrounding space, at both physiological and perceptual levels (Moseley et al., 2012). Some studies have attempted to modulate body distortions in patients suffering from brain damage using cross-modal illusions based on multisensory integration techniques and the “free-energy principle” (FEP; Friston et al., 2010; Limanowski and Blankenburg, 2013), through mirror visual feedback therapy (MVFT) and the rubber hand illusion (RHI; Bolognini et al., 2015; Tosi et al., 2018), or by manipulating visuo-tactile stimulation feedback in amputee patients presenting a telescoped effect (which occurs when the distal part of the phantom limb is perceived as shrinking within the stump; Schmalzl, 2011).

In this perspective article, we propose a new rehabilitation approach based on the use of full-body ownership illusions induced using a 360° video system designed to assess and later modulate the distorted internal body representation of the paretic upper limb in patients with stroke. The main aim of the proposed intervention is to provide kinesthetic and proprioceptive stimuli to patients with a paretic upper limb so that the internal representation of the affected limb changes from the distorted position to a normal one before starting conventional motor rehabilitation training. This intervention may help physicians to improve the outcome of motor rehabilitation. We additionally explore the current understanding of body perception and consequent distortions of internal upper-limb representations following a stroke injury. We then describe some cross-modal illusions used for upper-limb motor rehabilitation in patients with stroke such as the MVFT or the RHI. Finally, we discuss recent developments in virtual body ownership illusions using 360° video systems for the modulation of body representations.

## Body Perception Distortion and Motor Disruption in Patients with Stroke

It is known that negative plastic changes can occur in the brain after a stroke injury (Takeuchi and Izumi, 2012), affecting the patients’ functional mobility (Morone et al., 2018). Moreover, the disuse of the affected part of the body, such as the arm in patients with stroke presenting hemiparesis or hemiplegia of the body, can enhance negative plastic changes in the brain (Takeuchi and Izumi, 2012; Bassolino et al., 2014b). In particular, disuse of the upper limb following a brain injury can lead to a reduction of the cortical representation in motor and sensorimotor areas (Flor et al., 2006; Dohle et al., 2009; Bassolino et al., 2014a), further affecting the functionality of the affected limb. This process is commonly known as “learned paralysis” and has been investigated in humans in a study in which the author suggested that the no-movement visual feedback of the affected limb following a motor intention reinforces the acquired knowledge that the limb cannot move (Ramachandran, 1993). Then, the lack of movement of the affected limb results in a progressive shrinking of the representation of the affected limb in the somatosensory cortex (Ramachandran, 1993).

Besides the shrunken representation of the affected limb in the somatosensory cortex, other studies have reported that the lack of movement of the affected limb can lead to other types of body distortions in patients with stroke, such as the supernumerary phantom limb sensation (Bakheit and Roundhill, 2005)—the feeling of having an extra limb—which involves bilateral frontal, right parietotemporal cortices and the basal ganglia (Srivastava et al., 2008), or anosognosia—the denial of sensory, motor, or perceptual deficits in the paretic limb after brain injury—which occurs in patients with left brain damage (Berlucchi and Aglioti, 2010). In addition, patients with right-hemisphere brain damage presenting hemispatial neglect have a complex distortion of the body schema (e.g., ipsilesional deviation of the representation of the median sagittal axis of the body, or a bilateral narrowing in estimated body width; Rousseaux et al., 2014). Other studies have shown that brain damage affecting the motor system can lead to a disrupted awareness of motor actions, as well as of the control of such motor actions (Frith et al., 2000), as occurs in the alien hand syndrome (Biran and Chatterjee, 2004). Such disruptions can be associated with a distorted representation of the body or body parts in the brain (Frith et al., 2000). As a consequence of these sensorimotor alterations after brain damage, some studies have reported an impaired sense of ownership of the paretic limb in patients with stroke (Burin et al., 2015). Hence, based on the studies commented above, one may postulate that there is a link between alterations in internal models of body representation and motor awareness and motor control after suffering brain damage such as stroke. Such alterations of the internal models of the body might interfere with motor rehabilitation, for example, when using the MVFT due to mismatch between sensory signals and internal models of body representation.

## Cross-Modal Illusions in Neurorehabilitation: Mirror Therapy vs. Virtual Reality and 360° Video

In the last years, some researchers have proposed the use of cross-modal illusions for neurorehabilitation purposes (Bolognini et al., 2015), with the intention to regulate possible alterations of body representation in the brain and to restore motor ability after suffering brain damage. Cross-modal illusions occur when one sensory modality (e.g., touch) affects the experience of another sensory modality (e.g., vision). These illusions are not only mediated by inferential or higher-level cognitive processes, but also mediated by automatic multisensory interactions occurring at brain level (Bolognini et al., 2015). One example is the RHI, in which healthy participants experience the illusion of owning a rubber hand by receiving synchronous visuo-tactile stimulation to the real hidden hand and to the visible rubber hand (Botvinick and Cohen, 1998). One of the most well-known types of cross-modal illusions in the field of neurorehabilitation is MVFT, whereby the healthy limb of the patient is reflected in the mirror and, seeming visually superimposed on the location of the affected limb, it creates the illusion that the affected limb has recovered (Ramachandran et al., 2009 for a review). Then, when patients move their healthy limb, they have the illusion of moving their affected limb. Such movement illusions resulted in pain relief in patients with phantom limb pain, and in re-learning motor patterns in patients with stroke (Altschuler et al., 1999; Sathian et al., 2000; Garry et al., 2005; Chan et al., 2007; Sütbeyaz et al., 2007; Altschuler and Hu, 2008; Darnall, 2009; Ramachandran et al., 2009). Moreover, recent meta-analysis studies showed that motor visual feedback using MVFT may enhance motor rehabilitation outcome (Yang et al., 2018; Zeng et al., 2018). Even though cross-modal illusions are considered an attempt by the multisensory system to reconnect the affected sensory neural networks and bypass injured areas, these can be maladaptive when atypical or even when they are generated as a consequence of rearranged multisensory networks (Bolognini et al., 2013). One example of this is illustrated in a study conducted by Foell and colleagues, in which amputee patients with telescoped phantom limbs had an atypical illusory multisensory experience after completing MVFT, which did not cause any pain relief (Foell et al., 2014). Nevertheless, others have shown that it is possible to modify the distorted internal representation of the affected limb using MVFT in patients with stroke (Tosi et al., 2018). For instance, in the study conducted by Tosi et al. (2018), a forearm bisection task was specifically designed to measure the metric representation of the arm (i.e., its size). The results showed that after performing an MVFT session, bisection scores shifted distally from baseline, showing a partial correction of the distorted metric representation of that arm.

One explanation of the results obtained in the study by Foell et al. (2014) could be that the perceived internal distortion of the phantom limb influenced the vividness of the ownership illusion of the healthy limb reflected in the mirror. The mismatch between the internal distorted representation of the arm and the observed reflection of the arm in a normal position could reduce the feeling of ownership of the reflected arm, thus reducing the effectiveness of the therapy for pain relief. In this regard, the development of new augmented or VR systems, as well as the use of new 360° videos, through which it is possible to induce embodiment of a full virtual body observed from a first-person perspective (Maselli and Slater, 2013; Aitamurto et al., 2018), and the manipulation of morphological characteristics of the represented virtual body (Kilteni et al., 2012b; Serino et al., 2019), offers a potential alternative to the traditional MVFT (Rothgangel and Bekrater-Bodmann, 2019). In this line, a large number of studies have demonstrated that by changing the morphological characteristics of the represented body in VR, it is possible to modulate pain perception in healthy and clinical populations (Matamala-Gomez et al., 2019, 2020) and to improve motor performance in patients with stroke (Ambron et al., 2018). Hence, new VR or 360° video systems offer the possibility to fully reproduce the distorted internal representation of the affected body part while feeling embodied in a full virtual body before starting the rehabilitation process. One example of this was discussed by Turton and colleagues, where the authors presented a new digital media tool for communicating body perception disturbances through a virtual avatar in patients suffering from complex regional pain syndrome (Turton et al., 2013). The tool allowed the modification of a virtual avatar in terms of size, shape, and color, including the ability to lengthen or shorten limb segments, make them thicker or thinner, and even change the limb position to anatomically impossible positions, thus adapting the avatar’s limb position to the patient’s verbal description. Even though the effectiveness of MVFT to modulate body representation and to improve motor recovery after stroke has been largely demonstrated (Altschuler et al., 1999; Yavuzer et al., 2008; Michielsen et al., 2011; Rothgangel et al., 2011), there are still some physical limitations when representing the distorted internal representation of the body, which lead to a reduced sense of ownership of the observed limb and ultimately weaken the rehabilitation outcome. Here, a solution to tackle the mismatch between the sensory information provided and the internal models of body representation when using cross-modal illusions for motor rehabilitation is proposed.

## Increasing Mind-Body Communication Through 360° Videos to Enhance Motor Rehabilitation Outcome

Body ownership may rely on some degree of matching between internal models of the body and the experienced sensory feedback when using cross-modal illusions to induce BOIs. The use of cross-modal illusions for rehabilitation is made more difficult when people feel like the body they see (e.g., reflected in a mirror) is not in keeping with their internal representation. In this regard, the incorporation of new technologies such as VR to update the distorted body representation in clinical populations has been proposed before (Riva, 2008; Riva et al., 2017, 2019). These studies have shown a possible theoretical way to correct a dysfunctional representation of the body using virtual body ownership illusions and explain how a distorted self-perception within the FEP framework is the result of an inference process that minimizes prediction errors associated with self-perception (Friston et al., 2010; Limanowski and Blankenburg, 2013) More specifically, the FEP is based on the assumption that the brain implements hierarchical dynamical models to predict the causes of the processed sensory information (Friston and Kiebel, 2009; Bubic et al., 2010). Then, the FEP proposes that subjects can model their self-representation as a consequence of hierarchical predictive modeling, mediated by the sensory information arriving to the body (Friston, 2011, 2013). Conceptually, the FEP assesses the improbability (surprise) of the sensory information under a hierarchical generative model (Friston et al., 2006, 2010, 2011). In the case of body ownership illusions, altered body perception results from a self-representation that is updated dynamically by the brain to minimize sensory conflicts (i.e., the differences between the predictions about sensory data and the real sensory data at any level of the hierarchical model). Then, when using body ownership illusions our brain tries to minimize the “surprise” through the predictive coding scheme, when encountering a signal that was not predicted, it will generate prediction errors and will update the model in order to minimize the differences between the predictions about sensory data and the real sensory data at any level of the hierarchical model (e.g., synchronous visuo-tactile feedback in the RHI study; Friston and Kiebel, 2009; Bubic et al., 2010; Friston et al., 2010). Therefore, the subjects will update their internal body representation.

**Figure 1:**
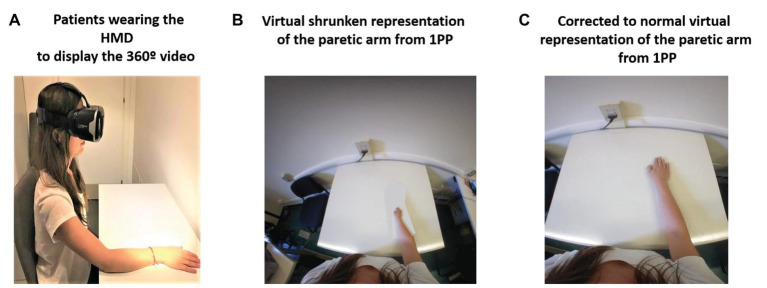
360° video protocol for managing body distortions in patients with stroke. Phase 1: **(A)** Patients wearing the head-mounted display (HMD) displaying the 360° video. **(B)** Virtual shrunken representation of the affected arm: Patients will observe the virtual body that will be colocated with their real body and will represent the patient’s described distorted representation of the upper limb from a first person-perspective (1PP; e.g., a shrunken upper limb). Phase 2: **(C)** First-person perspective observation of the progressive transformation of the affected upper limb from the distorted representation to a normal one through an edited 360° video viewed through the HMD.

Here, we propose a new rehabilitation approach by means of full-body ownership illusions recorded from a first-person perspective by a 360° video system and delivered through a head-mounted display (HMD) also from a first-person perspective. The use of 360° video to provide body ownership illusions from a first-person perspective has been previously shown (Aitamurto et al., 2018). Moreover, 360° videos have also been used in clinical populations with neurological disorders (Serino et al., 2017; Realdon et al., 2019). The intervention aims to reproduce internal upper-limb distortions in patients with stroke while they embody a virtual body by means of colocation of the real body with the virtual one. In this regard, colocation has been identified as a key component to provide body ownership illusions by itself without the need to induce synchronous visuo-tactile or visuo-motor correlations (Slater et al., 2010). The proposed intervention is composed of four different phases: (1) The “*baseline phase or distortion assessment phase*”: in this phase, patients will provide a verbal description of their distorted internal representation of the upper limb, and a picture of the body of the patients from a first-person perspective will be taken. (2) “*Phase 1 or the virtual embodiment phase*”: in this phase, patients will embody a virtual representation of their own body, which will be colocated with the real one and which will show the described upper-limb distortion from a first-person perspective through the HMD. Patients will be seated on a chair and in front of a table (same pose as the observed virtual body) and will be asked to place their real body in the same position in which the virtual body is so that they are colocated. Patients will observe their virtual body for at least 45–60 s to induce the ownership illusion of the virtual body. (3) “*Phase 2 or the normalization of the distorted upper-limb representation phase*”: once the embodiment phase is done by means of colocation, and the ownership illusion of the distorted virtual arm and the body is induced, patients will progressively observe how the position of the upper limb whose representation is distorted changes until it arrives at a normal position. Phases 1 and 2 are represented in Figure 1, where the upper-limb distortion is shown as a shrunken representation of the affected limb. (4) “*Phase 3 or the motor training phase*”: once the normalization of the distorted upper-limb representation is achieved, patients will perform their conventional motor rehabilitation training using either visual feedback techniques, such as MVFT or VR training, or conventional physical rehabilitation training. The rehabilitation approach proposed here allows the therapist to correct the internal body representation of the affected upper limb before starting motor rehabilitation, bringing them the opportunity to provide proper kinesthetic and proprioceptive feedback. Hence, we hypothesize that visual exposure to a corrected representation of the paretic limb through a virtual body ownership illusion generated in a 360° video should provide enough predictive errors, based on the FEP explained above, to update the distorted internal representation of the body to a normal one (Riva et al., 2017; Riva, 2018).

## Concluding Remarks

In conclusion, we suggest that full virtual body ownership illusions provided by 360° videos may bring new opportunities for the assessment and modulation of distorted upper-limb representations in patients with stroke. Moreover, if this intervention is applied before starting conventional motor rehabilitation training, this could provide proper kinesthetic and proprioceptive feedback to the affected upper limb. Whether or not the introduced approach is capable of enhancing treatment effects of MVFT for motor recovery after stroke, however, has to be explored in the future. The rehabilitation approach proposed here uses a “Positive Technology” approach (Wiederhold and Riva, 2012; Inghilleri et al., 2015; Riva et al., 2016), to build a potential bridge between basic research and clinical applications in the field of neurorehabilitation.

## Data Availability Statement

The original contributions presented in the study are included in the article/supplementary material, further inquiries can be directed to the corresponding author.

## Ethics Statement

Written informed consent was obtained from the individual(s) for the publication of any potentially identifiable images or data included in this article.

## Author Contributions

MM-G contributed to the conceptualization of the manuscript, bibliographic review, and writing. CM contributed to the bibliographic review and writing of the manuscript. PC contributed to the conceptualization and revision of the manuscript. EP and OR contributed to the bibliographic suggestions, conceptualization, and revision of the manuscript. FM and GR contributed to the supervision of the manuscript. All the authors approved the final version of the manuscript for submission.

### Conflict of Interest

The authors declare that the research was conducted in the absence of any commercial or financial relationships that could be construed as a potential conflict of interest.
